# Microscopic Evaluation of Crystallization Dynamics in 0.25% Levobupivacaine Mixed with Common Regional Anaesthesia Adjuvants: An *in vitro* Compatibility Study

**DOI:** 10.4274/TJAR.2026.262399

**Published:** 2026-10-30

**Authors:** Visolat Sharipova, Shoira Yuldosheva, Ivan Fokin

**Affiliations:** 1 Republican Scientific Centre of Emergency Medicine Department of Anaesthesiology and Reanimation, Tashkent, Uzbekistan

**Keywords:** Adjuvants, anaesthesia, crystallization, drug incompatibility, levobupivacaine, pharmaceutic

## Abstract

**Objective:**

To investigate the crystallization dynamics of 0.25% levobupivacaine when combined with common regional anaesthesia adjuvants.

**Methods:**

This *in vitro* study examined mixtures of 0.25% levobupivacaine with dexamethasone (20:1), 25% magnesium sulfate (20:1.5), dexmedetomidine (20:1), 8.4% sodium bicarbonate (10:1), and adrenaline (1:100,000). The study protocol was an *in vitro* investigation. Since no human or animal subjects were involved, the study was exempt from formal ethics committee approval. An institutional waiver was obtained from the Ministry of Health of the Republic of Uzbekistan Research Administration of the Republican Scientific Emergency Medical Centre (ref no: 03-05/119, date: 15.01.2026). All mixtures were incubated at 37 °C and examined under light microscopy (40× magnification) immediately, at 15 minutes, and at 1 hour. Crystallization onset, crystal count, and crystal size were quantified. For accurate dimensional analysis, a stage micrometer was used to calibrate the digital imaging software, and a calibrated scale bar was superimposed on each micrograph. Statistical analyses were performed using IBM SPSS Statistics for Windows, version 30.0 (IBM Corp., Armonk, NY, USA). Overall changes across the three matched assessment times were evaluated using the Friedman test. Significant omnibus results were followed by paired Wilcoxon signed-rank tests with Holm adjustment for multiple comparisons. All tests were two-sided, and a *P* value <0.05 was considered statistically significant.

**Results:**

Solutions containing sodium bicarbonate and dexamethasone demonstrated the earliest onset of crystallization. Sodium bicarbonate (10:1) produced immediate and massive precipitation. Levobupivacaine-dexamethasone mixtures showed progressive crystallization, with particle counts exceeding 100 per field for particles larger than 10 μm within 60 minutes. Adrenaline, magnesium sulfate, and dexmedetomidine showed significantly lower crystal densities than those of alkaline mixtures.

**Conclusion:**

Based on the present *in vitro* findings, adding either 8.4% sodium bicarbonate or dexamethasone to 0.25% levobupivacaine produced rapid, substantial crystallization, suggesting a physicochemical incompatibility under the tested conditions. These findings suggest that direct admixture of these agents with levobupivacaine in the same syringe should be approached with caution pending confirmation in clinical studies.

**Main Points**

• Sodium bicarbonate (8.4%) results in immediate, extensive crystallization when mixed with 0.25% levobupivacaine.

• Dexamethasone induces time-dependent crystal formation, with crystals exceeding 10 μm within 60 minutes.

• Adrenaline, magnesium sulfate, and dexmedetomidine exhibit little evidence of crystallization, indicating high physical stability.

*• In vitro* microscopic analysis is an essential pre-clinical tool for screening drug-drug incompatibilities.

## Introduction

In contemporary regional anaesthesia practice, clinicians increasingly rely on intermediate-long-acting local anesthetics, such as bupivacaine, ropivacaine, and liposomal formulations. Among these, levobupivacaine - the S-enantiomer of bupivacaine - is often the clinician’s first choice, primarily due to its more favorable safety profile regarding cardiovascular and neurological toxicity.^1^ Despite their effectiveness, a clinical challenge remains: the relatively brief duration of action of these agents. To address this, pharmacological additives known as adjuvants are integrated into anaesthesia protocols to accelerate the onset of action, extend the analgesic effect, and mitigate systemic risks.^2^ Adjuvants are now recognized as pivotal elements in multimodal analgesia strategies. Current clinical literature supports the use of various substances, including adrenaline, dexmedetomidine, magnesium sulfate, sodium bicarbonate (NaHCO_3_), clonidine, and dexamethasone, to optimize nerve blocks.^3^ For instance, Abanoub M et al.^4^ demonstrated that incorporating dexamethasone into a transversus abdominis plane block for emergency laparotomy significantly decreased postoperative opioid requirements compared to using local anesthetics alone (*P*=0.05). Similarly, research by Zeng et al.^5^ on 312 patients indicated that combining both dexamethasone and dexmedetomidine as adjuvants provided a longer analgesic window than using either agent individually. Furthermore, a meta-analysis encompassing 21 randomized trials confirmed that magnesium sulfate as an additive markedly prolonged block duration and improved pain scores on the visual analogue scale (VAS) (mean difference =114.35 minutes; 95% confidence interval: 89.31-139.88) and reduced pain intensity on VAS (mean difference =-1.54 points; 95% confidence interval: -2.56 to -0.53).^6^ Despite these clinical benefits, the physicochemical interactions between these adjuvants and local anesthetics —specifically the risk of crystallization—remain under-researched. Local anesthetics are weak bases; both bupivacaine and levobupivacaine have a pKa of 8.1 and limited solubility in water. When combined with substances that alter the local pH, such as the weakly basic corticosteroid dexamethasone, these anesthetics may precipitate as crystalline forms. This crystallization is primarily driven by the transition of the anesthetic into its non-ionized state in an alkaline milieu. Microscopic studies have revealed that mixing dexamethasone with local anesthetics can result in the formation of numerous crystals of varying sizes. Therefore, a thorough evaluation of the crystallization potential is mandatory before implementing these adjuvant combinations in spinal, epidural, or peripheral nerve blocks to ensure patient safety. The objective of this study was to investigate the effects of various adjuvants on the crystallization processes of 0.25% levobupivacaine.

## Methods

This study was an in vitro investigation. Since no human or animal subjects were involved, the study was exempt from formal ethics committee approval. An institutional waiver was obtained from the Ministry of Health of the Republic of Uzbekistan Research Administration of the Republican Scientific Emergency Medical Centre (reference no. 03-05/119, dated January 15, 2026). Informed consent was not applicable because no human participants were involved.

The study utilized levobupivacaine (trade name “Sofanest”, 0.25%, 20 mL, SP LLC “Jurabek Laboratories”, Uzbekistan), dexamethasone (4 mg dexamethasone sodium phosphate, “Radiks” LLC), adrenaline (FSUE “Moscow Endocrine Plant”), magnesium sulfate (SP LLC “Jurabek Laboratories”, Uzbekistan), and dexmedetomidine (EVER Pharma, Germany) as adjuvants. All the preparations were heated in a water bath at 37°C prior to mixing. Crystallization of adjuvants mixed with the local anesthetic was observed using a Primo Star light microscope (ZEISS, Germany).

A 20 mL solution of 0.25% levobupivacaine, heated to 37 °C, was mixed with dexamethasone solution at a 20:1 ratio, magnesium sulfate solution at a 20:1.5 ratio, dexmedetomidine solution at a 20:1 ratio, and adrenaline solution diluted to 1:100,000 at an equivalent ratio to levobupivacaine. These selected ratios represent the minimum clinically used doses of adjuvants added to local anesthetics. Additionally, NaHCO_3_ (8.4%) was tested at a 10:1 ratio. All microscopic preparations were maintained at a physiological temperature of 37 °C on a heated stage during the primary microscopic examinations at 15 minutes and 1 hour to ensure clinical relevance.

Prepared solutions (1 drop each) were placed on a glass slide, covered with a coverslip, and examined under a light microscope at 40× magnification. To simulate *in vivo *conditions and prevent artifactual crystallization caused by sample dehydration or low-temperature exposure over time, the microscopic preparations were maintained at a constant temperature of 37 °C using a thermal stage during the observation periods (15 minutes and 1 hour post-preparation). The mixtures were immediately transferred to slides for microscopic evaluation. Crystallization rates and the shape, number and size of formed crystals were quantified using advanced digital image analysis. To ensure the reliability and reproducibility of the data, multiple AI-based models (including OpenAI ChatGPT-4o, xAI Grok-3, Anthropic Claude 3.5, and Google Gemini 1.5 Pro) were employed to cross-verify the microscopic measurements. The final grade of crystallization (GoC) was determined via a “majority consensus” protocol (agreement of at least 3 models). In cases of split results, a senior investigator, blinded to the adjuvant type, performed the final assessment. Crystal size, shape, and count were quantified using a validated 0-5 point grading scale (Table 1).^7^

Microscopic images were captured using a Primo Star light microscope (ZEISS, Germany) equipped with a 40× objective lens. For accurate dimensional analysis, a stage micrometer was used to calibrate the digital imaging software. A 20-µm scale bar was superimposed on each micrograph to provide a consistent visual reference for crystal size and density across all time points.

### Statistical Analysis

Statistical analyses were performed using IBM SPSS Statistics for Windows, version 30.0 (IBM Corp., Armonk, NY, USA). For each adjuvant combination, 15 independently prepared replicates were evaluated; each replicate was assessed immediately after preparation and at 15 and 60 minutes. The independently prepared replicate mixture was considered the experimental unit. Continuous variables are presented as mean ± standard deviation, and the ordinal GoC score is reported as an ordinal score. Overall changes across the three matched assessment times were evaluated using the Friedman test. When the omnibus test was statistically significant, paired post-hoc comparisons were performed using Wilcoxon signed-rank tests with Holm adjustment for multiple comparisons. When crystal size could not be measured immediately because no crystals were detected, the 15- and 60-minute measurements were compared using the Wilcoxon signed-rank test. Outcomes that remained constant across all assessment times were not statistically tested, and crystal appearance and morphology were assessed descriptively. All tests were two-sided, and a *P* value <0.05 was considered statistically significant.

## Results

Levobupivacaine, an isomer of bupivacaine, is associated with a lower incidence of cardiotoxicity and neurotoxicity than bupivacaine. It has a pKa of 8.1 and a pH range of 4-6.5 and is prepared in an acidic environment to ensure its water solubility. The addition of agents that shift the pH of the solution toward alkalinity accelerates the crystallization of levobupivacaine.

Microscopic observation revealed that crystallization had commenced in the 20:1 mixture of 0.25% levobupivacaine and dexamethasone upon preparation. The number, size, and degree of crystallization of the formed crystals were highest compared with those in preparations in which other adjuvants were added to an equivalent dose of levobupivacaine (Figure 1).

In the first field of view (A), crystallization was observed to have commenced immediately after preparation, as revealed by microscopic examination. The second field of view (B) shows the crystallization process 15 minutes after preparation, while the third field of view (C) shows the microscopic analysis 1 hour after preparation. Quantitative parameters of the formed crystals, including the total count, mean size, average surface area, and GoC, were assessed using digital image processing. To ensure high analytical precision, multiple large language models with vision capabilities, OpenAI ChatGPT (GPT-4o), xAI Grok-3, Anthropic Claude 3.5 Sonnet, and Google Gemini 1.5 Pro, were used for independent analyses. The final data points presented in Table 2 represent the consensus results obtained through cross-verification and inter-model agreement.

Microscopic analysis demonstrated that in a 20:1 mixture of levobupivacaine and dexamethasone, the number of crystals, their length, and the degree of crystallization increased over time. This is attributable to the alkaline environment created by dexamethasone upon mixing and the subsequent decrease in temperature. In contrast, magnesium sulfate solution, which is weakly acidic, exhibited a delayed onset of crystallization compared to dexamethasone when added to levobupivacaine, and the formed crystals were significantly smaller (Figure 2A-C). Crystal analysis revealed the following results (Table 3).

Adrenaline, being chemically a weak base, undergoes rapid oxidation and dehydration in neutral or alkaline environments. To prevent this and ensure molecular stability, sodium bisulfite is added to maintain the pH between 2.2 and 5.0. Consequently, when adrenaline is added to levobupivacaine, the crystallization process occurs slowly. Microscopic examination immediately after preparation revealed no visible crystals. After the preparation was cooled to room temperature and examined 15 minutes later, crystallization was observed to have commenced at the peripheral edges of the preparation (Figure 3, Table 4).

Dexmedetomidine exhibits high solubility with a pKa of 7.1 and pH range of 4.5-7.0. When the sample was mixed with levobupivacaine at a 20:1 ratio and examined by microscopy, the crystallization process proceeded slowly, and the formed crystals were small (Figure 4, Table 5).

As with adrenaline, no crystallization elements were observed in dexmedetomidine when mixed with levobupivacaine at 37 °C. When the preparations were re-prepared at room temperature and examined by microscopy, fine granules (up to 10 per field of view) were identified (Table 5).

In levobupivacaine-dexmedetomidine solutions, the crystallization process was extremely slow, with the passage of time and decreases in temperature having minimal impact on crystal formation. Granule size remained <10 µm for 1 hour. The literature reports the use of NaHCO_3_ as an adjuvant. Analysis of its crystallization in the presence of local anesthetics (ropivacaine, bupivacaine) revealed extensive crystallization attributable to its basic nature (GoC score =5). However, insufficient data exist regarding its effect on levobupivacaine crystallization. Therefore, we investigated the degree of crystallization at room temperature of a 10:1 levobupivacaine: NaHCO_3_ solution (recommended adjuvant dose in the literature). Immediately upon mixing, a cloudy precipitate formed (Figure 5).

Microscopic examination of the solution revealed numerous large crystals (50-100 µm) that had aggregated to form substantial precipitates (Figure 6).

## Discussion

Our study demonstrated that the addition of NaHCO_3_ and dexamethasone to 0.25% levobupivacaine triggered rapid and substantial crystallization, whereas adrenaline and dexmedetomidine maintained high physical stability. Specifically, NaHCO_3_ (10:1) produced immediate, substantial precipitation visible to the naked eye (Figure 5), whereas dexamethasone induced time-dependent crystal growth exceeding 10 µm within 60 minutes. These results provide critical evidence regarding the physical stability risks of specific adjuvant-local anesthetic mixtures in regional anaesthesia.

Within the field of anaesthesiology, there is a notable scarcity of scientific literature addressing the physicochemical interactions and crystallization phenomena between local anesthetics and various additives. In particular, comprehensive data regarding the safety and clinical compatibility of integrating adjuvants with levobupivacaine remain inadequate. To date, research on crystallization has predominantly focused on the combinations of bupivacaine, ropivacaine, and lidocaine with agents such as NaHCO_3_, clonidine, and dexamethasone. In a 2024 doctoral study, Jopua M.A. investigated the crystallization patterns in mixtures of 0.33% ropivacaine and dexamethasone at a 20:2 ratio. Microscopic evaluation immediately following preparation revealed the presence of rod-like crystals with lengths varying between 19 and 70 µm. A subsequent assessment at the 15-minute mark showed a proliferation of these crystals, with their dimensions expanding to 37-108 µm. The researcher highlighted a critical clinical concern: these crystal structures significantly exceed the size of human blood components, such as erythrocytes (7-8 µm), leukocytes (4.5-20 µm), and platelets (2-4 µm). Consequently, the unintentional intravascular administration of such mixtures during regional anaesthesia procedures poses a substantial risk of embolic complications.^7^Our findings with levobupivacaine are consistent with this concern: crystals exceeded 10 µm almost immediately. In a review by Tow et al.,^8^ the physical implications of combining dexamethasone with ropivacaine and levobupivacaine were evaluated. The authors noted that mixing these local anesthetics with dexamethasone at a 20:2 ratio resulted in near-instantaneous crystallization. Furthermore, these crystalline structures were found to remain stable and unchanged over a 24-hour observation period.^8^

Dexamethasone has emerged as a widely adopted adjuvant in contemporary regional anaesthesia. Its clinical utility in enhancing the quality and prolonging the duration of nerve blocks, alongside its role in minimizing the systemic toxicity of local anesthetics, is well-supported by a robust body of evidence, including various meta-analyses and systematic reviews.^9^ Recent mechanistic evidence also underscores the need for caution when using dexamethasone as an adjuvant. Recent preclinical evidence suggests a need for heightened vigilance regarding the perineural application of dexamethasone. A systematic review by De Cassai et al.^10^ indicates that while dexamethasone independently lacks significant neurotoxic potential at therapeutic doses, its interaction with local anesthetics may enhance *in vitro* cytotoxic responses. Furthermore, the authors emphasized that current *in vivo* data—though not definitively linking the combination to long-term nerve damage—are still constrained by methodological limitations and narrow scope, necessitating careful consideration before routine co-administration.^10^ The semiquantitative analysis conducted by Hoerner et al.^11^ revealed that mixtures of dexamethasone and local anesthetics, such as mepivacaine, reached the highest crystallization intensity (grade 5 on a 0-5 scale) under microscopic evaluation. Their statistical assessment of the variables influencing this process identified a moderate positive correlation between the duration of the mixture and the degree of crystallization (*P*=0.57, *P*=0.0003). In contrast, the correlation between pH variations and the initiation of crystal growth was identified as weak and lacked statistical significance (*P*=-0.17, *P* > 0.05). This observation, reported in the 2025 study by Hoerner et al.,^11^ suggests that the temporal factor (duration of the mixture) may exert a more decisive influence on physical stability than alkalinity across specific local anesthetic-adjuvant combinations. Similar findings have been reported in other studies. When 50 local anesthetic-adjuvant mixtures were examined under microscopy, 26 preparations exhibited a crystallization grade >4, and ropivacaine was found to form more crystals compared to bupivacaine.^12^ In a comparative analysis of anesthetic-steroid interactions, Hwang et al.^13^ observed distinct crystallization patterns among different agents. Their results indicated that lidocaine-based mixtures remained free of crystal formation. In contrast, combining ropivacaine with betamethasone caused visible precipitates, whereas its mixture with dexamethasone produced fewer crystals ranging from 10 to 100 µm in size. Furthermore, the combination of bupivacaine and betamethasone resulted in the proliferation of numerous micro-crystals, typically measuring up to 10 µm.^13^ While Hwang et al.^13^ reported specific crystal dimensions for bupivacaine and ropivacaine, our study highlights unique patterns for levobupivacaine, suggesting that the S-enantiomer structure may influence the interaction with adjuvants differently. Investigations into the impact of diverse additives on the stability of bupivacaine and ropivacaine have identified NaHCO_3_ as the most potent inducer of crystallization. In a semiquantitative microscopic analysis by Hoerner et al.,^14^ mixtures containing NaHCO_3_ exhibited both the highest density and the most substantial dimensions of crystal formations compared to other studied adjuvants. Bhoi et al.^15^ conducted a microscopic examination of clinically used ratios of the local anesthetics ropivacaine and lidocaine with the adjuvants dexmedetomidine and dexamethasone to study their effects on crystallization. They observed that dexamethasone at doses of 2, 4, and 8 mg produced crystals of nearly identical size and shape. When dexmedetomidine was added as a second adjuvant to these preparations, an increase in the number of crystals and a reduction in crystal size were noted. According to the conclusions of this study, dexamethasone enhances crystallization starting from a 2 mg dose, and this process is independent of dexamethasone dosage.^15^

The clinical implications of these findings should be interpreted cautiously because the present study was conducted *in vitro* and did not evaluate patient outcomes. Based on these *in vitro* findings, levobupivacaine-sodium bicarbonate and levobupivacaine-dexamethasone mixtures exhibited physicochemical incompatibilities under the tested conditions. These observations suggest that direct admixture in the same syringe warrants caution; however, they do not establish a clinical contraindication to this practice, nor demonstrate the superiority of intravenous over perineural dexamethasone administration. Route-specific recommendations require clinical evidence beyond the scope of this study.

### Study Limitations

This study has several limitations that should be acknowledged. First, it was conducted as an *in vitro* laboratory analysis, which may not fully replicate the dynamic physiological conditions present in a clinical setting, such as body temperature, local tissue pH, and blood flow. Second, while microscopic crystallization was observed, the long-term clinical impact of these precipitates on nerve function or systemic safety was not directly evaluated *in vivo*. Further clinical studies are required to correlate these laboratory findings with patient outcomes and potential neurotoxic risks.

## Conclusion

Based on the present *in vitro* findings, the addition of NaHCO_3_ or dexamethasone to 0.25% levobupivacaine substantially influenced crystal formation under the tested conditions. NaHCO_3_ produced immediate and massive precipitation, whereas the levobupivacaine-dexamethasone mixture showed time-dependent crystal proliferation. These findings suggest that direct admixture of these agents with levobupivacaine in the same syringe may raise physicochemical compatibility concerns. Because routes of administration and clinical outcomes were not compared, no conclusion can be drawn regarding the clinical superiority of intravenous versus perineural administration of dexamethasone.

## Ethics

**Ethics Committee Approval:** Not applicable. This study is an in vitro laboratory investigation and did not involve human subjects or animal experiments.

**Informed Consent:** Not applicable. This in vitro laboratory study did not involve human participants.

## Figures and Tables

**Figure 1:**
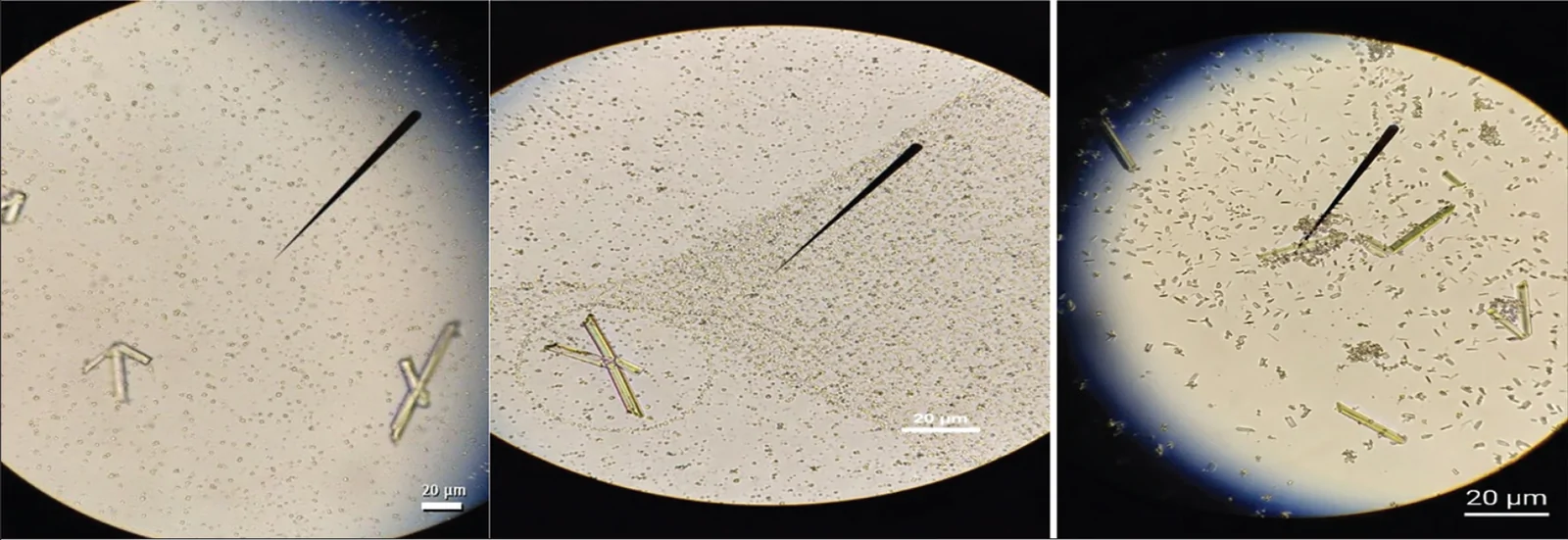
Microscopic appearance of crystals in 0.25% levobupivacaine and dexamethasone solution at a 20:1 ratio. A) Microscopic appearance of crystals immediately upon preparation; B) Microscopic appearance of crystals 15 minutes after preparation; C) Microscopic appearance of crystals 1 hour after preparation.

**Figure 2:**
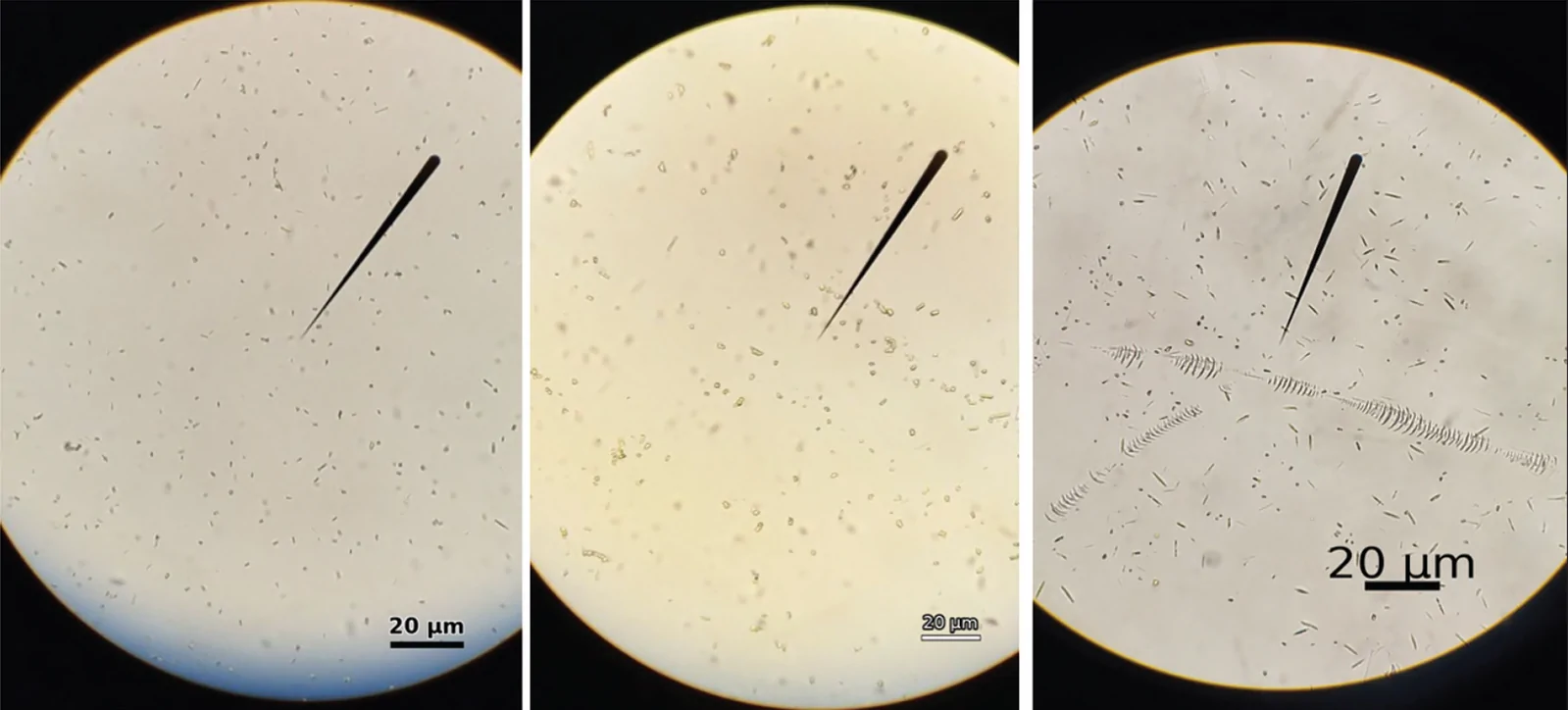
Microscopic appearance of crystals in 0.25% levobupivacaine and 25% magnesium sulfate solution at a 20:1.5 ratio. A) Microscopic appearance of crystals immediately upon preparation; B) Microscopic appearance of crystals 15 minutes after preparation; C) Microscopic appearance of crystals 1 hour after preparation.

**Figure 3:**
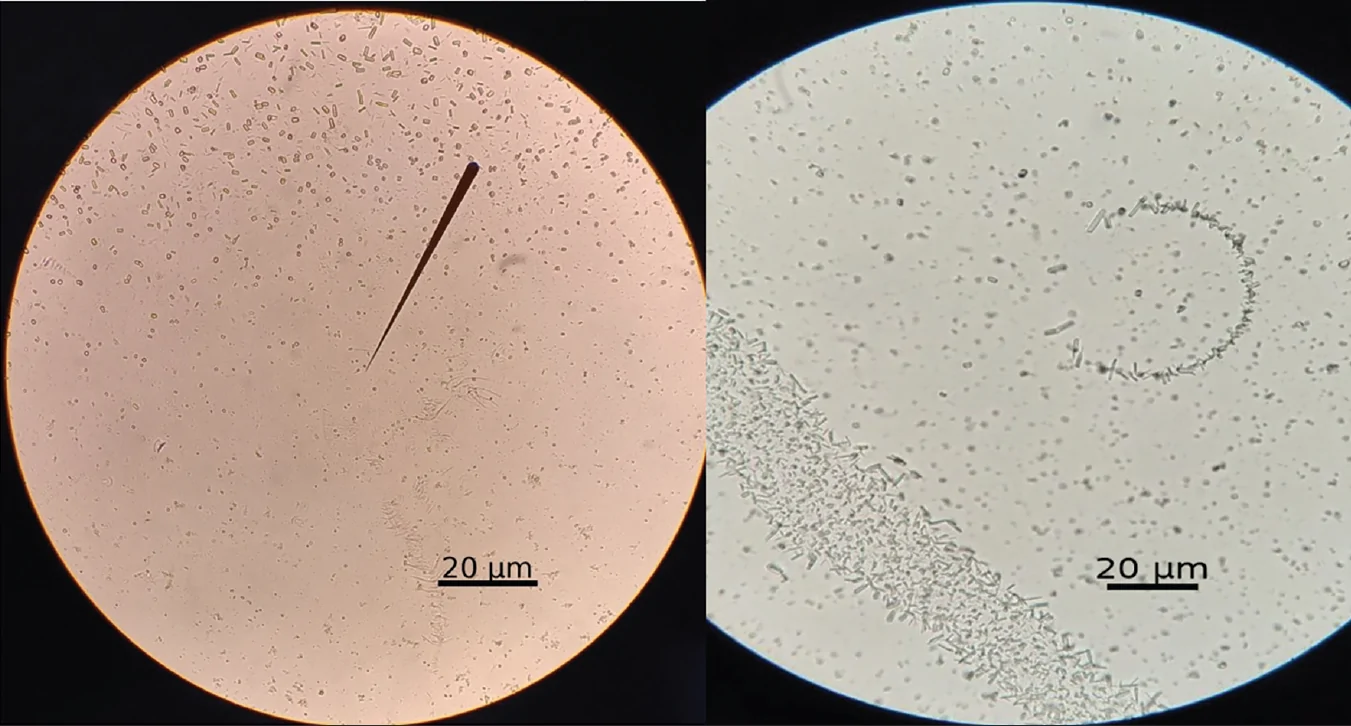
Microscopic appearance of crystals in 0.25% levobupivacaine mixed with adrenaline solution diluted to 1:100,000 ratio. A) Microscopic appearance of crystals 15 minutes after preparation; B) Microscopic appearance of crystals 1 hour after preparation.

**Figure 4:**
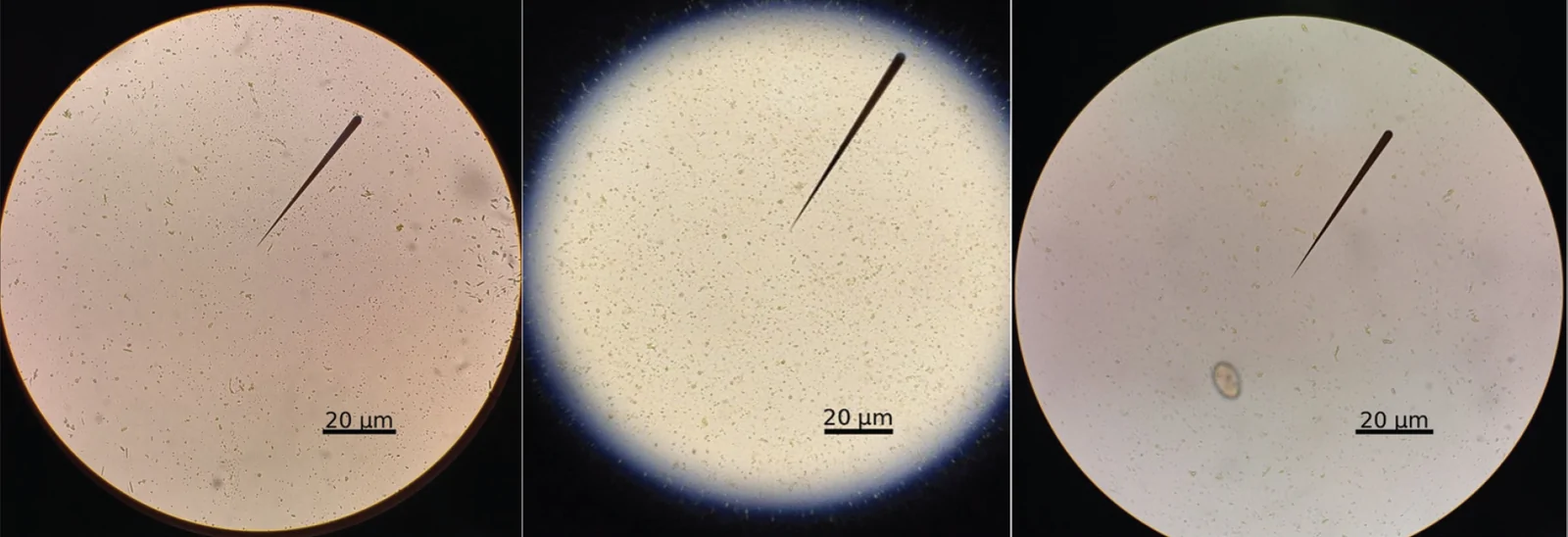
Microscopic appearance of crystals in 0.25% levobupivacaine mixed with dexmedetomidine at a 20:1 ratio. A) Microscopic appearance of crystals immediately upon preparation. B) Microscopic appearance of crystals 15 minutes after preparation; C) Microscopic appearance of crystals 1 hour after preparation.

**Figure 5:**
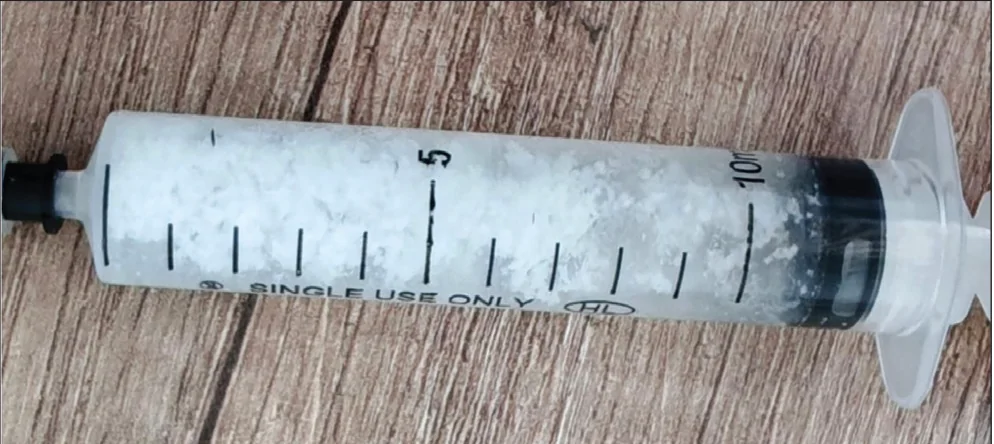
Precipitate formed upon mixing levobupivacaine with NaHCO_3_ at a 10:1 ratio. NaHCO_3_, sodium bicarbonate.

**Figure 6:**
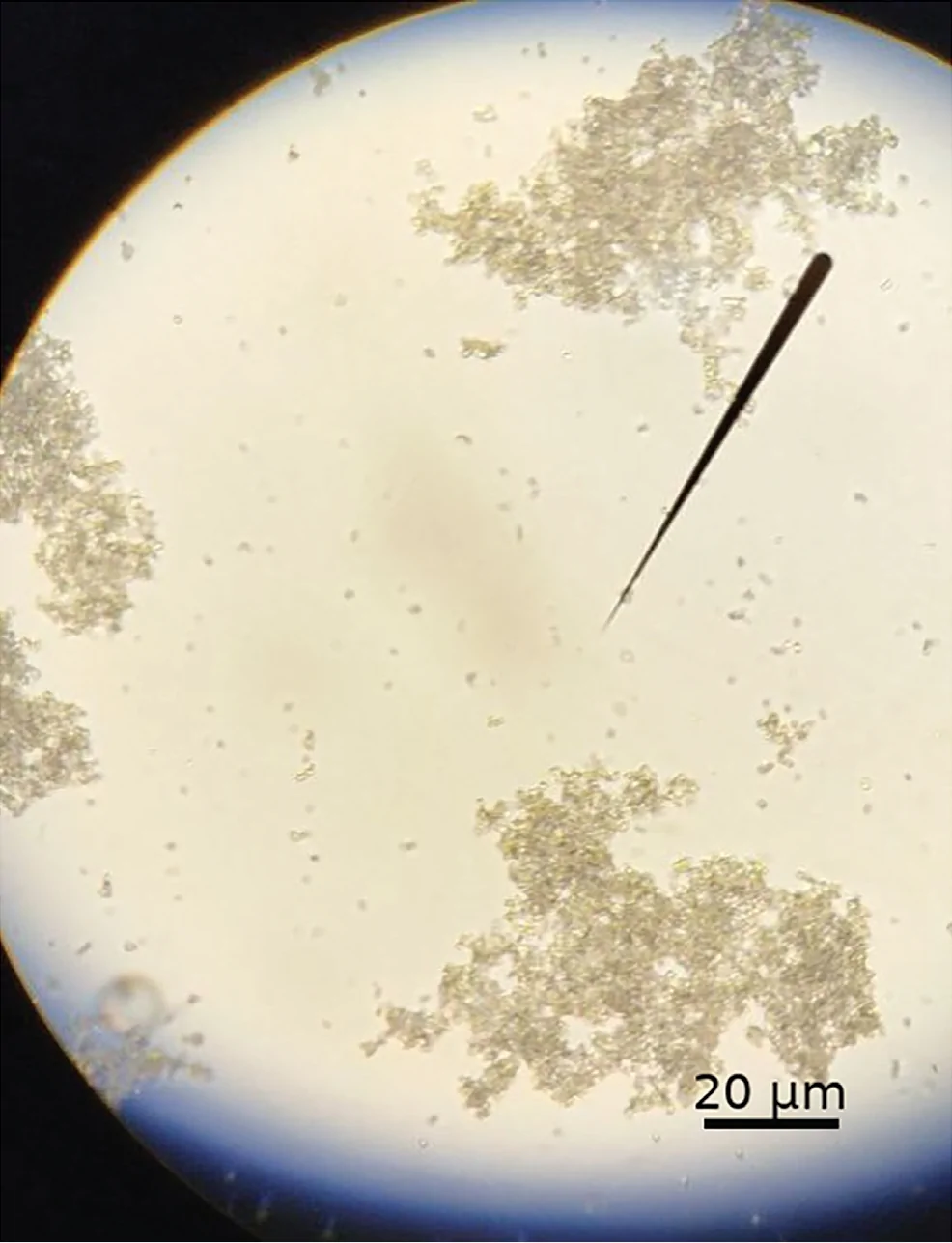
Microscopic appearance of the crystallization process in levobupivacaine and NaHCO_3_ solutions at a 10:1 ratio (immediately upon preparation). NaHCO_3_, sodium bicarbonate.

**Table 1. Crystallization Grading Scale (Adapted From)7:** 

**Grade**	**Description**
0	No crystals observed
1	1 crystal in 1 field of view, size >10 µm
2	2-10 crystals in 1 field of view, size >10 µm
3	>10 crystals (but <100) in 1 field of view, size >10 µm or 1 crystal, size >50 µm
4	>100 crystals in 1 field of view, size >10 µm or 1 crystal, size >50-<100 µm
5	>100 crystals in 1 field of view, size >10 µm and >1 crystal, size >50 µm and 1 crystal, size >100 µm

**Table 2. Analysis of Crystallization Process in 0.25% Levobupivacaine and Dexamethasone Solution at a 20:1 ratio at Respective Time Points:** 

**Parameters**	**Immediately upon preparation**	**15 minutes after preparation**	**1 hour after preparation**	***P* value**
**Crystal** **appearance,** **shape**	Small rod-shaped, clustered	Rod-shaped, clusters enlarged	Fully dispersed, rod- and plate-shaped	-
**Number** **of** **crystals** **in** **field** **of** **view** **(n)**	340±42 (n = 15)	630±56 (n = 15)	1275±89 (n = 15)	<0.001
**Mean** **crystal** **size** **(µm)**	9.1±1.2	12.3±1.5	17.4±2.1	<0.001
**Degree** **of** **crystallization** **(GoC** **score)**	4 (n = 15)	4 (n = 15)	4 (n = 15)	-

Note: Values for crystal count and crystal size are presented as mean ± standard deviation. The reported *P* values are omnibus *P* values from the Friedman test across the three matched assessment times. Significant omnibus results were followed by paired Wilcoxon signed-rank tests with Holm adjustment for multiple comparisons. The GoC score remained constant across all assessment times and was not statistically tested. Crystal morphology was assessed descriptively

GoC, grade of crystallization

**Table 3. Analysis of Crystallization Process in 0.25% Levobupivacaine and Magnesium Sulfate Solution at A 20:1.5 Ratio at Respective Time Points:** 

**Parameters**	**Immediately upon preparation**	**15 minutes after preparation**	**1 hour after preparation**	***P* value**
**Crystal** **appearance,** **shape**	Rare small crystals, scattered	Fine rod-shaped, minimal clustering	Small polyhedral crystals, dispersed	-
**Number** **of** **crystals** **in** **field** **of** **view** **(n)**	45±12 (n = 15)	82±18 (n = 15)	156±24 (n = 15)	<0.001
**Mean** **crystal** **size** **(µm)**	6.2±0.8	7.8±1.0	9.5±1.3	<0.001
**Degree** **of** **crystallization** **(GoC** **score)**	1 (n = 15)	2 (n = 15)	2 (n = 15)	<0.01

Note: Values for crystal count and crystal size are presented as mean ± standard deviation. The reported *P* values are omnibus *P* values from the Friedman test across the three matched assessment times. Significant omnibus results were followed by paired Wilcoxon signed-rank tests with Holm adjustment for multiple comparisons. Crystal morphology was assessed descriptively

GoC, grade of crystallization

**Table 4. Analysis of Crystallization Process in 0.25% Levobupivacaine Mixed with Adrenaline Solution Diluted To 1:100,000 Ratio at Respective Time Points:** 

**Parameters**	**Immediately upon preparation**	**15 minutes after preparation**	**1 hour after preparation**	***P* value**
**Crystal** **appearance,** **shape**	No crystals visible	Fine needle-like at periphery	Needle- and irregular-shaped, peripheral	-
**Number** **of** **crystals** **in** **field** **of** **view** **(n)**	0±0 (n = 15)	28±9 (n = 15)	67±15 (n = 15)	<0.001
**Mean** **crystal** **size** **(µm)**	N/A	5.4±0.7	7.2±1.0	<0.001
**Degree** **of** **crystallization** **(GoC** **score)**	0 (n = 15)	1 (n = 15)	2 (n = 15)	<0.001

Note: Values for crystal count and crystal size are presented as mean ± standard deviation. Overall temporal differences in crystal count and GoC score were evaluated using the Friedman test, followed by paired Wilcoxon signed-rank tests with Holm adjustment when the omnibus test was significant. Crystal size was compared between 15 and 60 minutes using the Wilcoxon signed-rank test because no crystals were detected immediately after preparation. Crystal morphology was assessed descriptively

GoC, grade of crystallization

**Table 5. Microscopic Analysis of Crystallization Process in 0.25% Levobupivacaine Mixed with Dexmedetomidine at A 20:1 Ratio:** 

**Parameters**	**Immediately upon preparation (37 °C)**	**15 minutes after preparation (RT)**	**1 hour after preparation (RT)**	***P* value**
**Crystal/granule** **appearance,** **shape**	No crystals/granules visible	Fine granules, scattered	Fine granules, minimal aggregation	-
**Number** **of** **crystals/granules** **in** **field** **of** **view** **(n)**	0±0 (n = 15)	8±3 (n = 15)	12±4 (n = 15)	<0.001
**Mean** **crystal/granule** **size** **(µm)**	N/A	3.2±0.5	4.1±0.6	<0.001
**Degree** **of** **crystallization** **(GoC** **score)**	0 (n = 15)	1 (n = 15)	1 (n = 15)	<0.01

Note: Values for crystal/granule count and size are presented as mean ± standard deviation. Overall temporal differences in crystal/granule count and GoC score were evaluated using the Friedman test, followed by paired Wilcoxon signed-rank tests with Holm adjustment when the omnibus test was significant. Crystal/granule size was compared between 15 and 60 minutes using the Wilcoxon signed-rank test because no crystals or granules were detected immediately after preparation. Morphology was assessed descriptively. Minimal crystallization compared to other adjuvants (*P* < 0.001); RT, room temperature (~22 °C)

GoC, grade of crystallization
